# Pharmacogenetics and the print media: what is the public told?

**DOI:** 10.1186/s12881-015-0172-3

**Published:** 2015-05-09

**Authors:** Basima Almomani, Ahmed F Hawwa, Nicola A Goodfellow, Jeffrey S Millership, James C McElnay

**Affiliations:** Clinical and Practice Research Group, School of Pharmacy, Medical Biology Centre, Queen’s University Belfast, 97 Lisburn Road, Belfast, BT9 7BL UK; Aston Pharmacy School, Aston University, Birmingham, B4 7ET UK

**Keywords:** Media, Newspapers, Pharmacogenetics, CNS

## Abstract

**Background:**

Pharmacogenetics is a rapidly growing field that aims to identify the genes that influence drug response. This science can be used as a powerful tool to tailor drug treatment to the genetic makeup of individuals. The present study explores the coverage of the topic of pharmacogenetics and its potential benefit in personalised medicine by the UK newsprint media.

**Methods:**

The LexisNexis database was used to identify and retrieve full text articles from the 10 highest circulation national daily newspapers and their Sunday equivalents in the UK. Content analysis of newspaper articles which referenced pharmacogenetic testing was carried out. A second researcher coded a random sample (21%) of newspaper articles to establish the inter-rater reliability of coding.

**Results:**

Of the 256 articles captured by the search terms, 96 articles (with pharmacogenetics as a major component) met the study inclusion criteria. The majority of articles over-stated the benefits of pharmacogenetic testing while paying less attention to the associated risks. Overall beneficial effects were mentioned 5.3 times more frequently than risks (p < 0.001). The most common illnesses for which pharmacogenetically based personalised medicine was discussed were cancer, cardiovascular disease and CNS diseases. Only 13% of newspaper articles that cited a specific scientific study mentioned this link in the article. There was a positive correlation between the size of the article and both the number of benefits and risks stated (P < 0.01).

**Conclusion:**

More comprehensive coverage of the area of personalised medicine within the print media is needed to inform public debate on the inclusion of pharmacogentic testing in routine practice.

## Background

The science of pharmacogenetics which aims to define the genes that affect our response to current drugs is a rapidly growing field that has gained enormous momentum due to recent advances in molecular genetics and genome sequencing [1]. It is clear now that much of the individuality in our response to medications is genetic in nature; understanding this individual variability defines the area of pharmacogenetics and can be a powerful tool to improve both the efficacy and safety of drug prescribing [2]. More recently, the term ‘pharmacogenomics’ has been introduced. While the latter term is broader and encompasses all genes that may determine drug response [3] the distinction between the two terms is arbitrary and both are often used interchangeably [4]. Not surprisingly, pharmacogenetic and pharmacogenomic discoveries are normally discussed in terms of their implementation into routine healthcare prompting the possibility of genetic testing to facilitate more effective drug therapy [5]. This has recently been acknowledged by the regulatory authorities (such as the US Food and Drug Administration, FDA and the European Medicines Agency, EMEA) with drug label revisions which include relevant pharmacogenetic information and published guidance on the use of clinical pharmacogenetics in early-phase clinical studies. Pharmacogenetics has therefore become one of the leading areas of personalised medicine with a potential to change the way in which healthcare is offered [6]. Despite its potential importance in clinical practice, little is known about how pharmacogenetics is portrayed in the news print media.

Recently, the analysis of media dialogue has been recognised in the literature as an important aspect within the area of health research [7]. Different media sources e.g. television, radio, internet, magazines and newspapers all share in conveying information to the public and special interest groups such as patients, healthcare professionals and policy makers [8,9]. The mass media have also been used as an important vehicle for communicating behavioural change to the public, with varying degrees of success [10-12]. A recent survey (Jan-Dec 2011) found that on average, 38.1% of adults in Great Britain had read at least one national daily newspaper within the previous day [13].

Stories about medicines in the media can be portrayed negatively (harms of medicine), positively (improved efficacy and safety) or may simply address recent findings and new research. Newspaper articles can play an important role in influencing demand for and provision of health services and in changing health related behaviour [9,14]. They can also create powerful changes in public opinion toward participation in research studies and policy discussions [7,15].

A number of studies which have investigated public and patient opinions about the provision of pharmacogenetics (PGx) testing have demonstrated support for the incorporation of PGx testing into patient management plans [16-19]. In recent years, the UK healthcare system has encouraged patient involvement in the decision making process regarding their healthcare [9]. Patient involvement in the decision making process in the area of current interest, however, implies that patients need to be properly informed about potential use of PGx testing in their treatment plans. Newsprint media play a key role in educating the public and shaping their knowledge, and therefore has the potential to influence opinion and attitudes toward such testing. As patients are becoming increasingly self-educated via the media [20], it was deemed important to evaluate how the print media portray information on PGx to the public. A number of questions are particularly pertinent, for example, has there been an increase in the number of newspaper articles on PGx over the last decade? Were the articles equally distributed between the different types of newspapers and hence broadly to the public? What potential benefits and risks of PGx testing have been highlighted in the newspaper stories and how were they framed to the public? In relation to PGx testing, which group of disease(s) or drug(s) are most commonly addressed by the print media?

The main aim of this study was therefore to explore, through systematic content analysis, the nature and trends in UK newspaper coverage of PGx in general and the potential benefits and risks of PGx testing in particular.

## Methods

**Figure 1:**
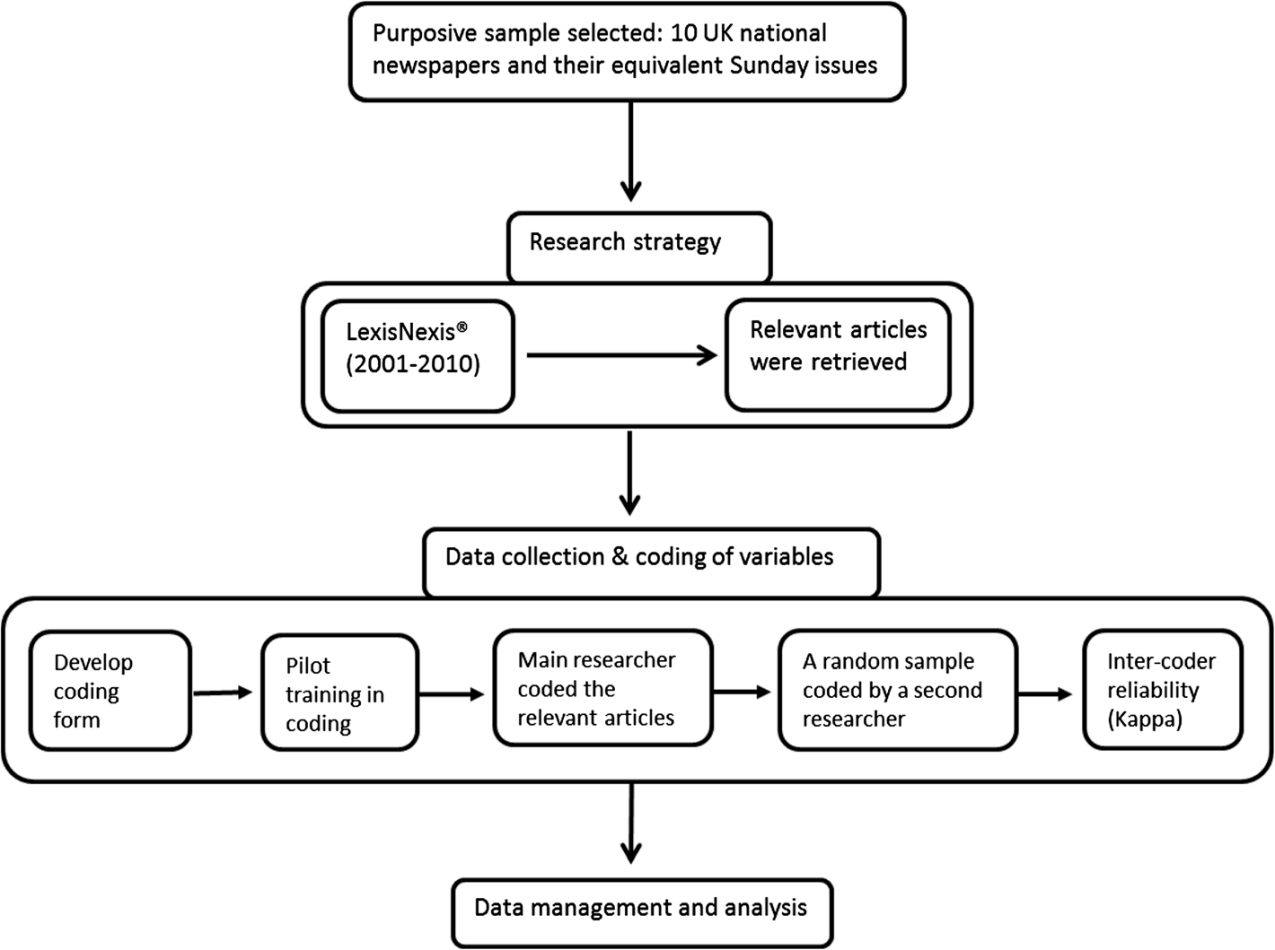
Overview of the content analysis methodology adopted.

We performed content analysis of the coverage of PGx in UK national newspapers published between 1^st^ Jan 2001 until 31^st^ Dec 2011. This timeframe was chosen to cover the most recent decade of news articles in the field. An overview of the methodological approach used in the present study is presented in Figure 1:

### Newspaper selection

The LexisNexis database [21] was used to obtain full text reprints of published newspaper articles from electronic archives. A purposive sample of 10 UK national daily newspapers and their Sunday equivalents was chosen. These newspapers were selected since they had the highest circulation figures at the time of initiating the study (May 2010) according to the Audit Bureau of Circulations [22] and provided a broad spread, of both ‘broadsheet’ to ‘tabloid’ outlets. In addition, two newspapers that have a high volume Sunday distribution (News of the World and The People) were included. The timeframe selected was 1^st^ Jan 2001 until 31^st^ Dec 2011. The UK national newspapers included in the study were as follows: *The Sun, Daily Mail (The Mail on Sunday), Daily Mirror (Sunday Mirror), Daily Star (Sunday Star), Daily Telegraph (Sunday Telegraph), Express (Sunday Express), Daily Record (Sunday Record), The Times (Sunday Times), Financial Times, The Guardian (The Observer), The News of the World and The People*.

### Search strategy and eligibility criteria

After empiric testing of different search terms, the keyword adopted in the search strategy was “pharmacogen!”. The “!” represented a wild card search and therefore the “pharmacogen!” search term encompassed pharmacogenetic(s) and pharmacogenomic(s). Both broad search terms were employed to minimise loss of relevant articles.

We set criteria for the inclusion and exclusion of retrieved articles for detailed analysis. Initial inclusion criteria included articles that contained any reference to PGx (in humans), including articles in any format (e.g. news article, editorial, letter to editor, etc...). Articles were retrieved if the search term was captured in the story headline, lead paragraph or body of the text. Retrieved articles were then subjected to more stringent exclusion criteria which removed articles that emphasised another topic with only an indirect reference to PGx (e.g. articles that contained only a brief mention of PGx; <10% of the article), articles that dealt solely with business issues (e.g. manufacturers’ share prices) or focused on PGx in the context of non-pharmacological products (e.g. cosmetic or diet products). If the same article appeared in both the daily and Sunday editions, the duplicate was removed; in such cases, the article with the higher word count was included in the analysis.

### Data extraction and coding frame

A standardised coding frame was developed, taking into account the published literature and discussions within the research team. To help ensure reliable coding, the coding instrument included a series of topics with standardised categorical responses which consisted of three main sections:

- Bibliographic information: the name of newspaper, year of publication, positioning in the newspaper, the length of the article and author name.
- Article contents: assessment of benefit/advantages, potential harm/risks, barriers to the application of PGx, medicine/disease/gene related to PGx, source of information and main commentator.
- Judgment and rating: we recorded additional variables including article slant “the skew of the report written about the event” i.e. weighting of benefits versus risks [23], article claim “Main message conveyed to public” i.e. whether exaggerated, balanced or understated when compared with conventional knowledge on the topic, and finally the quality of information [24]. The quality of information was assigned a subjective rating on a scale of 1–10 and then classified as poor (1–3), average/good (4–7) or excellent (8–10). An example of a poor quality article may only have contained a short statement about PGx or reported inaccurate information on PGx testing, whereas an article classified as high-quality would have included, for example, definitive information about PGx, have a scientific article as information source or included information about barriers or facilitators of PGx testing. In the case of newspaper articles linked to a scientific journal publication, a fourth section was applied to collect and compare the information presented in the two sources, in particular whether links with industry were cited and whether conflicts of interests were disclosed. Subjective rating of the main claim as exaggerated, balanced or understated was made by an expert coder trained in the field of PGx (BA) with reference to scientific articles in the field. In the case of indecision, other authors were consulted and a consensus reached on rating of the article.

### Data collection and coding of variables

Two independent researchers (pharmacists; BA, NG) were employed in the coding process. In order to familiarise the coders with the coding process, a pilot exercise was conducted on a sample of the articles; n = 10). Minor revisions were made post pilot as required after discussion with the research team.

Having completed the pilot, one researcher (BA) coded the entire set of relevant newspaper articles, while to provide a quantitative estimate of overall reliability of the coding process, a second researcher (NG) independently assessed a random sample (21%) of the articles utilising a web-based random number generator. The inter-coder agreement on categorical variables was tested using Kappa scores.

### Outcome measures and data analysis

Following data collection, all responses were coded and entered into SPSS (version 18, SPSS Inc, USA). Descriptive statistics (frequencies) were used to summarise the data for the total sample. Differences in the reporting of articles from different sources (tabloid versus broadsheet) and in the number of scientific articles published compared with reports in newspaper articles were carried out using a Chi square (χ^2^) test or Fisher Exact test as appropriate. The Wilcoxon signed-rank test was used to calculate the difference in the mean number of times benefits and risks were mentioned. Spearman’s correlation coefficient (Spearman’s rho r) was used to examine the relationship between word counts and the number of benefits and risks stated in the articles. The Mann–Whitney *U*-test (non-parametric) was also used where appropriate. Statistical significance was set at p ≤ 0.05.

## Results

### Inter-coder agreement

The rate of agreement between coders on dichotomous variables was high e.g. whether benefits and risks were mentioned in the article, however, when variables required greater subjective judgement, such as claim and quality of the information, lesser agreement was achieved. Kappa scores across all coding variables ranged from 0.57 to 1, indicating moderate to perfect agreement for all of the candidate variables [25] (Table 1).

**Table 1:** **Inter-coder agreement on items within the coding frame**

**Question (number of choices)**	**Observed agreement**	**Kappa value**
Main themes (8 choices)	90%	0.88
Key perspectives (7 choices)	90%	0.79
Benefit stated (yes/no)	100%	1.0
Risk stated (yes/no)	100%	1.0
Barrier stated (yes/no)	90%	0.79
Quotation stated (yes/no)	100%	1.0
Source of information (5 choices)	95%	0.91
Main voice (4 choices)	95%	0.89
Article slant (4 choices)	100%	1.0
Main claim (3 choices)	85%	0.58
Quality of information (3 choices)	80%	0.57

### Frequency of reporting

A total of 256 articles mentioning PGx appeared in the high circulation UK newspapers evaluated between Jan 2001- Dec 2011. Of the 256 articles captured by the search terms, 26 duplicate articles were omitted, by selecting the articles with the higher word counts. Of the remaining 230 articles, 96 articles were retained after applying the stricter exclusion criteria and thus were included in the final detailed analysis, although the 230 articles were also included in a number of interim analyses, e.g. exploring the pattern of annual frequency of reporting over time and differences in the reporting of relevant articles in different newspaper types. This minimised loss of relevant information pertaining to articles excluded using the stricter inclusion/exclusion criteria employed. A total of 11 articles were directly linked to eight scientific journal papers. The mean word count for the 96 articles was 590.5 words and 38 (40%) of these articles appeared in the main news sections.

**Figure 2:**
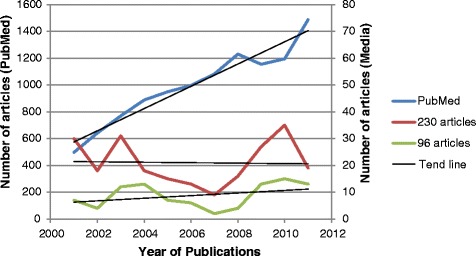
Number of articles that mentioned PGx in the UK national newspapers studied superimposed with PubMed articles containing PGx as a key word.

The pattern of the annual frequency of appearance of articles distributed across the period was quite similar whether for the 230 investigated articles or for the 96 articles selected for detailed analysis (Figure 2). To allow comparison with scientific publications over the period, the frequency of articles included in PubMed® has also been included in this figure. A breakdown of the articles by newspaper showed that of the 230 articles, 216 (94%) were published in daily newspapers, i.e. only 6% were from Sunday newspapers. PGx articles occurred most frequently in the Financial Times (32%), the Times (25%) and the Guardian (13%). No articles about PGx appeared in the Sunday Mirror, the Sunday Star, the Sunday Record, the News of the World or the People newspapers.

We found that many more articles pertaining to PGx were published in the broadsheet newspapers compared with the tabloid newspapers (85% vs 15% of the 96 articles; P < 0.001). However, over time, the number of articles published in the tabloids increased, i.e. 22% of the articles were published in the second half of the investigated period (July 2006–2011) compared to 7% in the first half (2001- June 2006) (P = 0.04).

### Content of the news stories selected for detailed investigation

**Figure 3:**
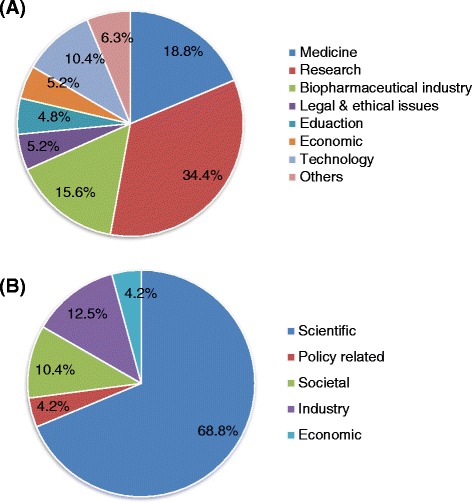
The distribution of PGx articles (n = 96) according to **(A)** the main themes covered and **(B)** the key perspectives addressed.

Within the 96 articles selected for detailed investigation, the main themes covered were research (34%), medicines (19%) and the biopharmaceutical industry (16%; Figure 3A). Over half of the articles (55%) covered other themes in addition to the main area of discussion. The key perspective in the majority of articles was scientific (69%; Figure 3B).

**Figure 4:**
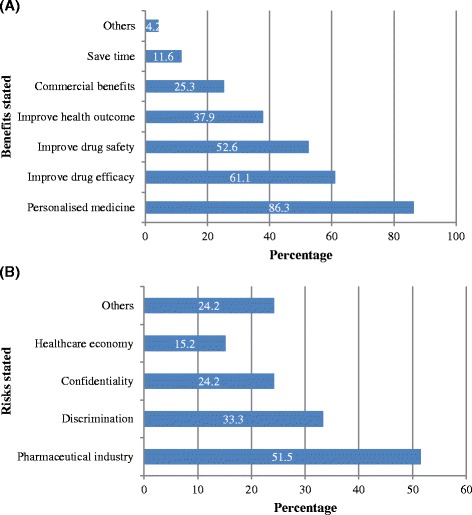
Reported benefits **(A)** and risks **(B)** associated with PGx testing (n = 96 articles).

Out of the 96 included articles, 95 (99%) stated at least one benefit for the application of PGx. The total number of benefits identified was 267 (range 1 to 7 per article). Of those articles that qualified the potential benefit, 82 (86%) reported that using PGx based approaches/testing would help to establish personalised medicine, 58 (61%) reported improving drug efficacy and 50 (53%) reported improving drug safety (Figure 4A). In contrast, only 33 articles (34%) mentioned at least one risk associated with PGx application; the total number of risks highlighted was 50 (range 1 to 3 per article). Of those articles that detailed potential risks, 17 (51%) reported that PGx would adversely affect the economic stability of the pharmaceutical industry, 11 articles (33%) reported a risk of discrimination being introduced into medical treatment and 8 articles (24%) reported the risk of loss of privacy and confidentiality of genetic data (Figure 4B). Importantly, no article mentioned a risk without listing at least one benefit. There was a significant difference (p < 0.001) between the mean number of times benefits (2.78) and risks (0.52) were stated in individual articles. There were significantly positive correlations between the length of the articles (word counts) and number of benefits (Spearman’s rho r = 0.32, p = 0.001) and number of risks (Spearman’s rho r = 0.331, p = 0.002) stated.

**Figure 5:**
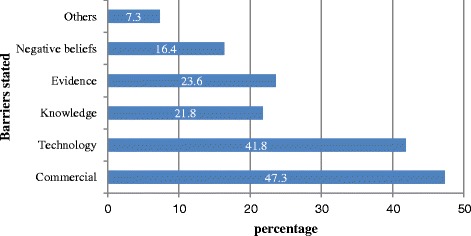
Reported barriers to adoption of PGx testing (n = 96 articles).

More than half of articles (57%; n = 55) reported potential barriers to adopting PGx testing into routine practice. The total number of barriers identified was 92 (the mean number of barriers was 0.96, range 1 to 5 per article). Of those articles that qualified the nature of the barriers, 47% reported commercial factors and 42% reported technology factors (Figure 5).

Seventy five articles (78%) reported disease related PGx items, 64 articles (67%) reported medicine related PGx items and 37 (39%) of them reported gene related PGx items. Of those articles that discussed disease related PGx topics, the majority focused on chronic diseases such as cancer (mainly breast cancer), cardiovascular diseases (CVD) and diseases of the central nervous system (CNS). Medicine related PGx topics focused mainly on, anticancer drugs whether licensed such as Herceptin® (trastuzumab) or unlicensed at the time of publication such as Iressa® (gefitinib), Tarceva®, (erlotinib) or Velcade® (bortezomib). Furthermore, of those articles that covered gene related PGx topics, the most common gene discussed was the human epidural growth factor receptor 2 (HER2) gene followed by liver enzyme genes.

Over three quarters (77%; n = 74) of the articles cited the source of information and in these scientists (73%) and scientific journals (15%) were the main sources quoted. With regards to the main voice, commentators from different disciplines (academic, scientific or industry spokesperson) were the main voice for 60% (n = 58) of the articles while journalists were the main voice for the remainder. The majority of the articles (79%; n = 76) cited at least one expert and the total number of commentators mentioned was 167. Eleven scientific articles were referred to and in eight of these scientific articles there was an acknowledgement of links with industry. Only one newspaper article mentioned the link with industry.

### Judgement of news stories

The majority of articles (66%; n = 63) were categorised as having a positive slant, a third (33%; n = 32) were categorised as having a mixed slant and only one article was categorised as having a neutral slant. The majority (66%; n = 63) of the articles were categorised as having a balanced claim, 29% (n = 28) were categorised as having an exaggerated claim and 5% (n = 5) of articles were categorised as having an understated claim. More than half (59%; n = 57) of the articles were categorised as having good information, a fifth (19%; n = 18) were categorised as having excellent information and 22% (n = 21) were categorised as having poor information. There was a significant positive correlation between the overall size of the articles (word count) and the quality of the information contained in the articles (Spearman’s rho r = 0.62, p <0.001), i.e. longer articles tended to be of higher quality.

Articles with the journalist as the main voice were more likely to report the risk of PGx testing (p = 0.03) and consequently demonstrated a less positive slant (p = 0.01). On the other hand, those articles with the journalist as the main voice were less likely to include quotations from experts (p < 0.001) and to use scientific papers as sources of information (p < 0.001). A detailed comparison of the articles with scientists and journalists as the main voices is presented in Table 2.

**Table 2:** **Comparison of articles with scientists and journalists as the main commentator**

**Variable**	**Scientist main voice (n = 58)**	**Journalist main voice (n = 38)**	**P value**
**Mean number of words per article**	605.8	567.1	NS
**Benefit stated**	58 (100%)	37 (97.4%)	NS
**Mean number of benefits stated**	2.97	2.50	NS
**Risk stated**	15 (25.9%)	18 (47.4%)	**0.03**
**Mean number of risks stated**	0.38	0.74	**0.03**
**Barrier stated**	33 (56.9%)	22 (57.9%)	NS
**Mean number of barriers stated**	0.91	1.0	NS
**Articles with quotation from expert**	53 (91.4%)	23 (60.5%)	**<0.001**
**Mean number of quotes per article**	2.14	1.16	**<0.001**
**Slant (positive)**	44 (75.9%)	19 (50%)	**0.01**
**Claim (exaggerated)**	18 (31%)	10 (26.3%)	NS
**Quality of information (good)**	34 (58.6%)	23 (60.5%)	NS
**Source of information (scientific papers)**	10 (17.2%)	1 (2.6%)	**<0.001**

## Discussion

The medium of newspapers was selected in this study for various reasons: firstly, newspapers are one of the most popular UK media sources and have a wide range of readership. Secondly, it was possible to easily access and search published articles and address, for example, how information was presented during specific periods [23]. Thirdly, there is evidence that “newspaper coverage is strongly correlated with radio and television reporting on similar issues” [23].

The results of this study revealed that PGx is a topic of only marginal interest to UK newspaper editors as only 230 articles mentioned PGx over the 11 year period within the higher readership newspapers included in the study. Of 96 articles included in the detailed analysis, only 11 of them (15%) were linked to scientific papers. Similarly, the work of Bubela *et al.* [26] which involved a comparison of genetic research covered in 26 newspapers from four countries over a seven year period (Jan 1995- June 2001) with scientific papers found only 2% (14 articles) from a total of 626 articles had a PGx focus.

There was a marked unequal distribution of newspaper articles addressing PGx in the broadsheet (85.4%) vs tabloid (14.6%) press. A plausible explanation is that editors of tabloid newspapers targeted less complex material for their readership. This, however, has the potential to significantly impact the sections of the public that are exposed to this type of material. In contrast, Hilton and Hunt [27] reported that more articles about the swine flu pandemic appeared in the tabloids than in the ‘serious’ and middle market UK newspapers.

### Content of the news stories

In the current study, it was noted that the majority of articles emphasised the benefits of PGx testing while paying less attention to potential risks, i.e. only 33 articles (34%) mentioned the risks compared to 95 articles (99%) that mentioned the benefits. Interestingly, beneficial effects (267 in total) were mentioned 5.3 times more frequently than risks (50 in total). This trend of reporting is consistent with other studies that reported media analyses on health topics. For example, Bubela and Caulfield [26] in their comparison of newspaper articles and scientific papers about genetic research reported that only 15% of newspaper articles and an even smaller number of scientific papers (5%) discussed risks. Similarly, Cassels *et al.* [28] reported that 68% of Canadian newspaper articles did not mention a single potential harm associated with five new medications. Many researchers have commented on such errors of exclusion [7,29,30]. Moynihan *et al.* [29] suggested that journalists’ behaviour in reporting the positive effects might reflect their enthusiasm to hype a certain topic, which could be viewed as potentially misleading. Conversely, journalists have also been keen to hype harmful effects in certain situations. For example, Bartlett *et al.* [31] reported that newspapers were more likely to sensationlise and hype bad news emanating from medical research when compared to press releases. Also, Schwartz and Woloshin [32] reported that most of news media coverage of mammography screening, and Tamoxifen for the prevention of breast cancer, emphasised the risks.

It was noted in the present study that articles with the journalist as the main voice were more likely to report risks compared to those articles with the scientist as the main voice. Reasons for this are unclear but could be due to journalists wishing to report both sides of the story i.e. risks and benefits; whereas scientists tended not to consider the advances made by them as carrying any particular risks.

A high quality report should provide the reader with a “balanced assessment” so that informed decisions can be made [33,34]. If benefits are overstated, the public expectation for cure or health improvement may be inflated and unrealistically raised [3]. The opposite is also an issue i.e. if a risk is overstated, this may generate unnecessary anxiety which could impact adversely on patient behaviour [9].

There were significant positive correlations between the size of articles (word count) and both the number of benefits and risks stated in the articles. Consistent with this, Holtzman *et al.* [29] identified that incompleteness of information presented in many of the articles published in the US media about the discoveries related to genetic disease was partly due to the short length of the articles. They found a significant positive correlation between the length of article and its content score. This highlights the importance of article length in comprehensively reporting the significant issues on a particular topic.

In the present study, seven out of eight articles that cited a scientific paper in which the researchers had links with industry, failed to report that potential conflict of interest. This finding is supported by work of Moynihan *et al.* [29] and Bubela *et al.* [30] who found that the majority of newspaper articles did not mention links with industry (61%), and potential conflicts of interest (95%) respectively, that had been detailed in published scientific papers. Also, Holtzman *et al.* [35] found that only 5% of the 228 articles they researched mentioned potential conflict of interest, although the majority of journalists thought it to be an essential item in story reporting. These findings highlight the fact that the media pay less attention to conflicts of interest disclosed, a factor that could easily lead to misinterpretation of research findings [7,9,23]. Interestingly, McComas and Simone [36], who conducted an analysis of media articles on conflict of interest in science between 1992–2001, found that there was a steady trend in the number of publications on conflict of interest over the 10 year period, with a peak in 2000.

In the present study, the most frequently reported diseases/medicines/genes that were related to PGx issues were in the field of cancer. This finding highlights that advances in the area of PGx are most notable in the oncology setting, where Herceptin® and other anticancer drugs that can be selected based on the patient genetic profile, are used [37].

### Judgement of news stories

Of the 96 papers, 63 articles about PGx were presented to the public with a positive slant (66%), no articles were skewed negatively and 1 article (1%) was presented with a neutral slant. The remainder of the articles were mixed, i.e. referred to PGx as being both beneficial and harmful. This is not surprising as the two variables used in determining the category of slant were the reporting of benefits and risks and in the majority of cases the benefits were emphasised and the risks were under-reported. In contrast, Stebbing *et al.* [23] found that about 71% of news articles about patient safety were presented with a neutral slant. In addition, 28 articles in this study (29%) were presented with an exaggerated claim. Consistent with this, Bubela and Caulfield [26] reported that a third of articles about genetic research were categorised as having an exaggerated claim.

There are some limitations to the generalisability of the present study findings since the analysis was limited to higher circulation newspapers, however, circulation figures can only be estimated based on the number of newspaper sold and not on the actual readership. Additionally, the newspaper database itself has limitations as complete coverage of every article is not always achieved due to copyright restrictions [23]. Finally, newsprint media was the only media surveyed in the present study; other mass media news sources such as television, radio and the ‘web’ are also important sources of health information to the public. There is a strong correlation, however, between news items covered by the range of mass media formats [38].

## Conclusions

Newsprint coverage of public health issues, such as PGx and personalised medicine, are an important vehicle in delivering health messages to the public and in changing perceptions and behaviours. Journalists therefore have special responsibilities in conveying accurate and unbiased information about health and medicine, and should take into account that the reader might make important decisions based on what is presented [28,30,39]. As suggested by others [29] journalists should be more aware of advancements in health and science and carefully consider the evidence available, with particular attention to the following points: (i) the type of benefit; (ii) the type of risk and costs; (iii) groups of patients that can be helped and (iv) potential conflict of interest. Such an approach has the potential to improve the overall quality of media coverage. Scientists also carry the responsibility in the first instance for making important discoveries accessible to newspaper editors via press releases and secondly in ensuring such press releases are balanced in their claims.
